# Supplementary material for: Origin of ocean island basalts in the West African passive margin without mantle plume involvement
Source: Nat Commun. 2019 Jul 9;10:3022. doi: 10.1038/s41467-019-10832-7 (PMC6616360; doi:10.1038/s41467-019-10832-7)
Supplement: Supplementary file 1 — Supplementary Information [file 41467_2019_10832_MOESM1_ESM.pdf]

Supplementary Information

Origin of ocean island basalts in the West African passive margin without mantle plume involvement

Belay et al.

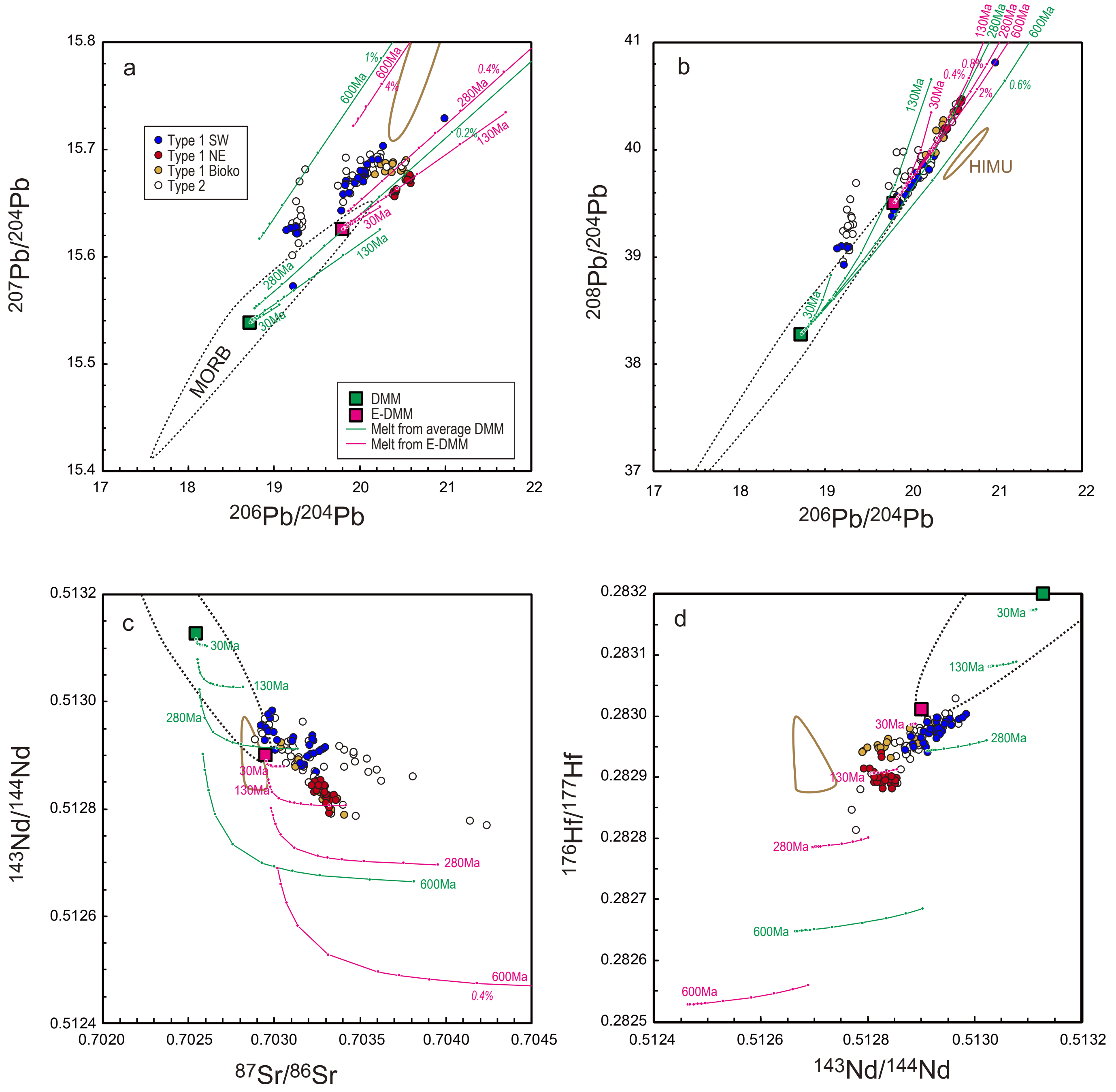

Supplementary Figure 1 | The calculated Sr, Nd, Hf, and Pb isotopic evolution curves of the ancient asthenospheric mantle-derived silicate melt at various ages. **a,** $^{206}Pb/^{204}Pb$ vs. $^{207}Pb/^{204}Pb$, **b,** $^{206}Pb/^{204}Pb$ vs. $^{208}Pb/^{204}Pb$, **c,** $^{87}Sr/^{86}Sr$ vs. $^{143}Nd/^{144}Nd$, and, **d,** $^{143}Nd/^{144}Nd$ vs. $^{176}Hf/^{177}Hf$. Sticks in each curve show the different degree of partial melting: 0.1, 0.2, 0.4, 0.6, 0.8, 1, 2, 4, 6, 8, and 10%. Range shown with brown is for HIMU basalts from St. Helena[1].

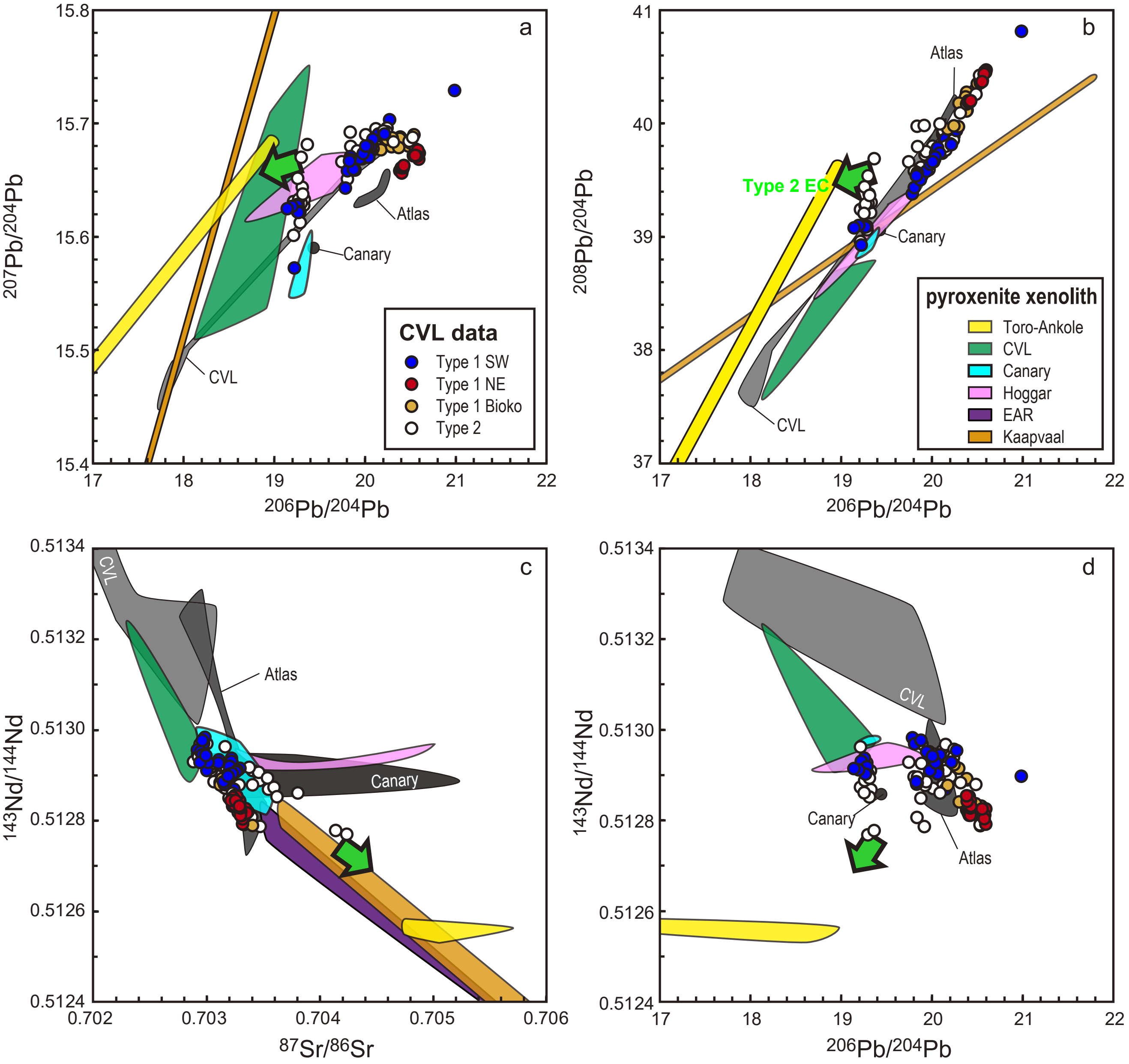

Supplementary Figure 2 | Sr-Nd-Pb isotopic compositions of the mantle peridotite from WAIB-IB and pyroxenite xenoliths from Africa compiled from GEOROC database. **a**, vs. $^{207}Pb/^{204}Pb$, **b,** $^{206}Pb/^{204}Pb$ vs. $^{208}Pb/^{204}Pb$, **c,** $^{87}Sr/^{86}Sr$ vs. $^{143}Nd/^{144}Nd$, and, **d,** $^{206}Pb/^{204}Pb$ vs. $^{143}Nd/^{144}Nd$. Colour regions indicate pyroxenite xenolith from the CVL[2,3], the Atlas Mountains[2,4-6], the Hogger Swell[7], the Canary Islands[8-11], the East African Rift (EAR)[12-16], the Tanganyika Rift (Toro-Ankole region)[17], and the Kaapvaal craton[18,19]. Grey regions indicate peridotite xenolith and clinopyroxene separates in peridotite xenolith from the CVL[3], the Canary Islands[8,9,11], and the Atlas mountains[5,6,20]. The lime green-coloured arrow indicates the direction of Type 2 EC. The source data of the compiled values for pyroxenite are provided in ref. 21. All the Pb isotopic data for Atlas samples were influenced by carbonatitic melt metasomatism[20].

Supplementary References

1 Stracke, A. Earth's heterogeneous mantle: A product of convection-driven interaction between crust and mantle. *Chem. Geol.* **330–331**, 274-299, (2012).

2 France, L. *et al.* Mantle refertilization and magmatism in old orogenic regions: The role of late-orogenic pyroxenites. *Lithos* **232**, 49-75, (2015).

3 Lee, D.-C. *et al.* Melt enrichment of shallow depleted mantle: a detailed petrological, trace element and isotopic study of mantle-derived xenoliths and megacrysts from the Cameroon line. *J. Petrol.* **37**, 415-441, (1996).

4 Pezzali, I., France, L., Chazot, G. & Vannucci, R. Analogues of exhumed pyroxenite layers in the Alboran domain sampled as xenoliths by Middle Atlas Cenozoic volcanism. *Lithos* **230**, 184-188, (2015).

5 Raffone, N. *et al.* Metasomatism in the Lithospheric Mantle beneath Middle Atlas (Morocco) and the Origin of Fe- and Mg-rich Wehrlites. *J. Petrol.* **50**, 197-249, (2009).

6 Natali, C. *et al.* Carbonated alkali-silicate metasomatism in the North Africa lithosphere: Evidence from Middle Atlas spinel-lherzolites, Morocco. *Journal of South American Earth Sciences* **41**, 113-121, (2013).

7 Kaczmarek, M. A. *et al.* Metasomatized Mantle Xenoliths as a Record of the Lithospheric Mantle Evolution of the Northern Edge of the Ahaggar Swell, In Teria (Algeria). *J. Petrol.* **57**, 345-382, (2016).

8 Whitehouse, M. J. & Neumann, E. R. Sr-Nd-Pb isotope data for ultramafic xenoliths from Hierro, Canary Islands: Melt infiltration processes in the upper mantle. *Contrib. Mineral. Petrol.* **119**, 239-246, (1995).

9 Vance, D., Stone, J. O. H. & O'Nions, R. K. He, Sr and Nd isotopes in xenoliths from Hawaii and other oceanic islands. *Earth Planet. Sci. Lett.* **96**, 147-160, (1989).

10 Neumann, E. R., Wulff-Pedersen, E., Pearson, N. J. & Spencer, E. A. Mantle Xenoliths from Tenerife (Canary Islands): Evidence for Reactions between Mantle Peridotites and Silicic Carbonatite Melts inducing Ca Metasomatism. *J. Petrol.* **43**, 825-857, (2002).

11 Neumann, E. R. *et al.* Serpentinization and Deserpentinization Reactions in the Upper Mantle beneath Fuerteventura Revealed by Peridotite Xenoliths with Fibrous Orthopyroxene and Mottled Olivine. *J. Petrol.* **56**, 3-31, (2015).

12 Teklay, M., Scherer, E. E., Mezger, K. & Danyushevsky, L. Geochemical characteristics and Sr–Nd–Hf isotope compositions of mantle xenoliths and host basalts from Assab, Eritrea: implications for the composition and thermal structure of the lithosphere beneath the Afar Depression. *Contrib. Mineral. Petrol.* **159**, 731-751, (2010).

13 Cohen, R. S., Onions, R. K. & Dawson, J. B. Isotope geochemistry of xenoliths from East Africa: Implications for development of mantle reservoirs and their interaction. *Earth Planet. Sci. Lett.* **68**, 209-220, (1984).

14 Bell, K. & Dawson, J. B. in *Carbonatite Volcanism: Oldoinyo Lengai and the Petrogenesis of Natrocarbonatites* (eds Keith Bell & Jörg Keller) 100-112 (Springer Berlin Heidelberg, 1995).

15 Koornneef, J. M. *et al.* Nature and timing of multiple metasomatic events in the sub-cratonic lithosphere beneath Labait, Tanzania. *Lithos* **112, Supplement 2**, 896-912, (2009).

16 Rudnick, R. L., McDonough, W. F. & Chappell, B. W. Carbonatite metasomatism in the northern Tanzanian mantle: petrographic and geochemical characteristics. *Earth Planet. Sci, Lett.* **114**, 463-475, (1993).

17 Davies, G. R. & Lloyd, F. E. Pb–Sr–Nd isotope and trace element data bearing on the origin of the potassic subcontinental lithosphere beneath south-west Uganda. *Kimberlites and Related Rocks, 2, Geological Society of Australia Special Publication* **14**, 784-794, (1989).

18 Rosenbaum, J. M. Mantle phlogopite: a significant lead repository? *Chem. Geol.* **106**, 475-483, (1993).

19 Viljoen, K. S., Schulze, D. J. & Quadling, A. G. Contrasting Group I and Group II Eclogite Xenolith Petrogenesis: Petrological, Trace Element and Isotopic Evidence from Eclogite, Garnet-Websterite and Alkremite Xenoliths in the Kaalvallei Kimberlite, South Africa. *J. Petrol.* **46**, 2059-2090, (2005).

20 Wittig, N. *et al.* Tracing the metasomatic and magmatic evolution of continental mantle roots with Sr, Nd, Hf and and Pb isotopes: A case study of Middle Atlas (Morocco) peridotite xenoliths. *Geoch. Cosmoch. Acta* **74**, 1417-1435, (2010).

21 Belay, I. G. *Geochemical study of the Cameroon Volcanic Line: Implication for the genesis of passive margin intraplate magmatism.* PhD thesis, Okayama University, <http://eprints.lib.okayama-u.ac.jp/en/55565>, (2017).
