# Supplementary material for: Origin of ocean island basalts in the West African passive margin without mantle plume involvement
Source: Nat Commun. 2019 Jul 9;10:3022. doi: 10.1038/s41467-019-10832-7 (PMC6616360; doi:10.1038/s41467-019-10832-7)
Supplement: Supplementary file 3 — Description of Additional Supplementary Files [file 41467_2019_10832_MOESM3_ESM.pdf]

Description of Additional Supplementary Files

File Name: Supplementary Data 1

Description: Major, trace, and Sr-Nd-Hf-Pb isotopic compositions of CVL lavas
